# Transmission networks of long-term and short-term knowledge in a foraging society

**DOI:** 10.1093/pnasnexus/pgaf258

**Published:** 2025-09-08

**Authors:** Haneul Jang, Daniel Redhead

**Affiliations:** Institute for Advanced Study in Toulouse, Toulouse School of Economics, 1 Esplanade de l’Université, Toulouse cedex 06 31080, France; Department of Human Behavior, Ecology and Culture, Max Planck Institute for Evolutionary Anthropology, Deutscher Platz 6, Leipzig 04103, Germany; Department of Human Behavior, Ecology and Culture, Max Planck Institute for Evolutionary Anthropology, Deutscher Platz 6, Leipzig 04103, Germany; Department of Sociology, University of Groningen, Grote Rozenstraat 31, Groningen 9712 TG, The Netherlands; Inter-University Center for Social Science Theory and Methodology, University of Groningen, Groningen 9712 TG, The Netherlands

**Keywords:** cultural transmission, cooperation, social networks, foraging skills, food location information

## Abstract

Cultural transmission across generations is key to cumulative cultural evolution. While several mechanisms—such as vertical, horizontal, and oblique transmission—have been studied for decades, how these mechanisms change across the life course, beyond childhood, remains unclear. Furthermore, it is under-explored whether different mechanisms apply to distinct learning processes: long-term learning—where individuals invest time and effort to acquire skills—and short-term learning—where individuals share information of immediate use. To investigate the network structure of these two types of knowledge transmission—long-term learning of foraging skills and short-term learning of food location information—we present social network data (1,633 nominations) collected from all inhabitants (aged 4 to 75) of a BaYaka community in the Republic of the Congo. Applying latent network models that estimate and adjust for measurement biases typical to self-reported data, we find that the demographic structure of a population—age distribution, sex, kinship, and marriage—shapes the dynamics of community-wide knowledge transmission. Foraging skills are transmitted within smaller, sparser networks with limited reciprocity, whereas food location information is exchanged more widely and reciprocally among peers. Both long-term and short-term knowledge transmission extend into adulthood, with adults learning from older adults, peers, and marital partners, and sharing knowledge with younger generations. Crucially, individuals tend to report more accurately about the partners with whom they shared knowledge than about those from whom they received knowledge. Our findings provide important empirical evidence on how community-wide cultural transmission is structured by demography and perception, and how these factors operate across different learning processes in a contemporary foraging society.

Significance Statement Transmission of foraging skills and food location information within communities is critical for daily cooperation and long-term survival, especially in foraging societies. Combining data from a forager community with social network analyses, we show that foraging skills are transmitted from older to younger generations, whereas food location information is transmitted more broadly and reciprocally across a community. Adults continue to learn from elders, peers, and spouses, and bridge knowledge transmission between younger and older generations. Individuals recall to whom they share knowledge more accurately than from whom they receive it, suggesting that cognitive biases influence knowledge transmission dynamics. This study demonstrates how demographic structure shapes community-wide knowledge-sharing networks and uncovers distinct patterns in long-term and short-term learning processes.

## Introduction

Humans depend on cultural transmission—the transfer of information between individuals and across generations—for successful adaptation to diverse environments (1–4). Theory in cultural evolution has emphasized the roles of several mechanisms that guide how and when individuals learn (5) and shape who individuals learn from (6–8). Recent formal theoretical (i.e. computational or mathematical) models have also shown that cultural learning strategies can vary depending on the type of information being transmitted (9) and environmental conditions (10), and that they are also influenced by cognitive factors such as fallible memory (11). In this study, we categorize cultural learning into two types of learning processes: long-term learning, where individuals acquire skills that take time and effort to develop (e.g. foraging techniques), and short-term learning, where individuals rapidly acquire practical, context-specific information (e.g. food locations). While long-term learning enables the accumulation of cultural knowledge across generations, short-term learning allows for quick adaptation to environmental or social changes. Examining the network structure of these two types of learning within a foraging population provides the opportunity to identify the ways in which distinct types of knowledge are transmitted within a community, providing a more nuanced understanding of how cultural transmission can be structured in diverse ways based on the content of the cultural information.

Long-term cultural learning can typically occur through three primary pathways: vertical, horizontal, and oblique transmission (12) (see Ref. (13) for expanded categories). In the multistage learning model, these pathways evolve over the course of childhood: children begin by learning from their parents (i.e. vertical transmission), then gradually expand their knowledge by engaging with peers (i.e. horizontal transmission) and other knowledgeable adults (i.e. oblique transmission) (9, 12–18). In foraging societies, where subsistence knowledge and skills are essential, childhood represents a critical period for acquiring ecological knowledge and subsistence-related skills (19, 20). Children often begin learning from their parents (21–23) and by practicing these skills through play with peers (24–28). Adolescents learn more complex tasks, such as hunting and tool-making, from skilled nonkin adults (14, 16, 17, 21, 29, 30). Learning, however, does not end in adolescence; it continues throughout life, as many complex skills—such as hunting, honey collecting, as well as ethnobiological knowledge—require years to refine and master (19, 20, 31–34). Despite this, most empirical research on hunter–gatherer cultural learning has focused on children (e.g. 13, 17, 24, 25, 30, 35–37), with few studies examining adult learning (see Refs. (22, 38, 39)). Given this, it remains unclear from whom individuals learn, and how this changes across the life course—from early childhood to late adulthood—particularly from a network perspective, which is crucial for understanding cultural learning in hunter–gatherers (40 ).

In addition to long-term learning of foraging skills, learning the locations of food resources is crucial. In foraging societies, individuals cooperate in subsistence activities and share food to reduce daily variability in food returns (41–44 ). They also exchange information about food locations, especially temporary sources, such as mushroom or wild yam patches, fruiting trees, honey trees, or animal nests. Central-place foraging, a key feature of hunter–gatherer social organization, facilitates this exchange, as foragers return to a central location after their trips (45). Information sharing relies on prior learning and shared experience of forest ecology and spatial environments, and is particularly effective in humans due to our ability to quickly convey and interpret complex messages through language (46). Despite its importance, it remains an open question whether such short-term learning is structured by the same mechanisms as long-term learning, such as preferences for learning from close kin (47), same-sex individuals (48), or specific age-structured learning (49 ), as predicted by cultural evolutionary models.

A recent theoretical model distinguishes between age-structured learning strategies in long-term and short-term processes. The “copy-the-old” strategy, favoring experienced individuals, is advantageous in stable environments where skills are hard to acquire but crucial for survival. In contrast, “copy-the-young,” which integrates individual learning and social information, supports rapid adaptation in changing environments (10). Based on this, short-term knowledge (e.g. food locations) can be exchanged more flexibly, while long-term, difficult-to-acquire skills (e.g. foraging techniques) are better suited to “copy-the-old.” Beyond age, the types of knowledge can also shape transmission mechanisms, such as biases toward kin, spouses, and same-sex individuals. Empirical research shows that broad community sharing occurs for food plants and social norms, whereas medicinal plant knowledge is more selectively passed between spouses and close kin (50 ). Additionally, same-sex learning biases exist, favoring men in male-dominated domains (e.g. fishing) and women in female-dominated ones (e.g. medicinal plants) (51 ). However, a gap remains in our understanding of how these demographic factors influence the transmission of short-term knowledge and how it compares to that of long-term knowledge.

Here, we investigate the mechanisms that shape community-wide knowledge transmission networks in a BaYaka forager community in the Republic of the Congo. The BaYaka are a group of Congo Basin forest foragers who engage in hunting and gathering for daily food acquisition, alongside small-scale crop cultivation (52, 53). Consequently, learning foraging techniques and skills during childhood is crucial (35, 54). The BaYaka often go on foraging trips with a group of other individuals (55) and share food to mitigate the risks of variable foraging returns (56). As such, sharing information about food locations is a critical component of their cooperative subsistence activities. We surveyed all 132 BaYaka inhabitants (aged from 4 to 75) of the village, asking them self-reported, directed, and double-sampled network questions. These questions focused on with whom they shared important foraging skills and techniques (i.e. long-term knowledge transmission) and with whom they shared food location information (i.e. short-term knowledge transmission).

To examine whether these two distinct knowledge transmission networks exhibit unique structural patterns, we compare how kinship (47), sex-based homophily, marital partnership, age structure, and reciprocity (57, 58) characterize these networks. First, we predict that kinship plays an important role in knowledge transmission networks for both foraging skills and information about food locations. However, we expect that nonkin friendships are also important (see Ref. (59)), particularly when sharing information about food locations rather than foraging skills. Second, given the sexual division of labor typical to many foraging societies (60, 61), we predict that sex-based homophily will structure knowledge transmission networks: females are more likely to share foraging skills and information on food locations with other females, whereas males are likely to share with other males. Third, we also predict that marital partnerships will facilitate knowledge sharing, particularly of food locations. In foraging societies, men and women engage in complementary subsistence activities—i.e. men primarily hunt animals, while women predominantly gather more reliable food sources, such as plants (60, 62–65 ). In such contexts, husbands and wives would share not only food at the end of the day but would also exchange information about food locations that they encounter during subsistence activities. Fourth, we expect that foraging skill-sharing networks will be more influenced by age structure, reflecting the stability of adaptive foraging knowledge across generations. In contrast, food location-sharing networks are expected to be more flexible across age groups, where reciprocity and mutual information exchange play a crucial role. Lastly, in light of recent theories suggesting that cultural transmission is influenced by memory biases (11), we further explore whether individuals perceive their knowledge-sharing partners differently depending on whether they are sending or receiving knowledge. By examining these dynamics, we provide empirical insights from a contemporary foraging community, contributing to a broader understanding of how demographic factors and individual perceptions shape both long-term and short-term knowledge transmission networks.

## Results

**Fig. 1.**
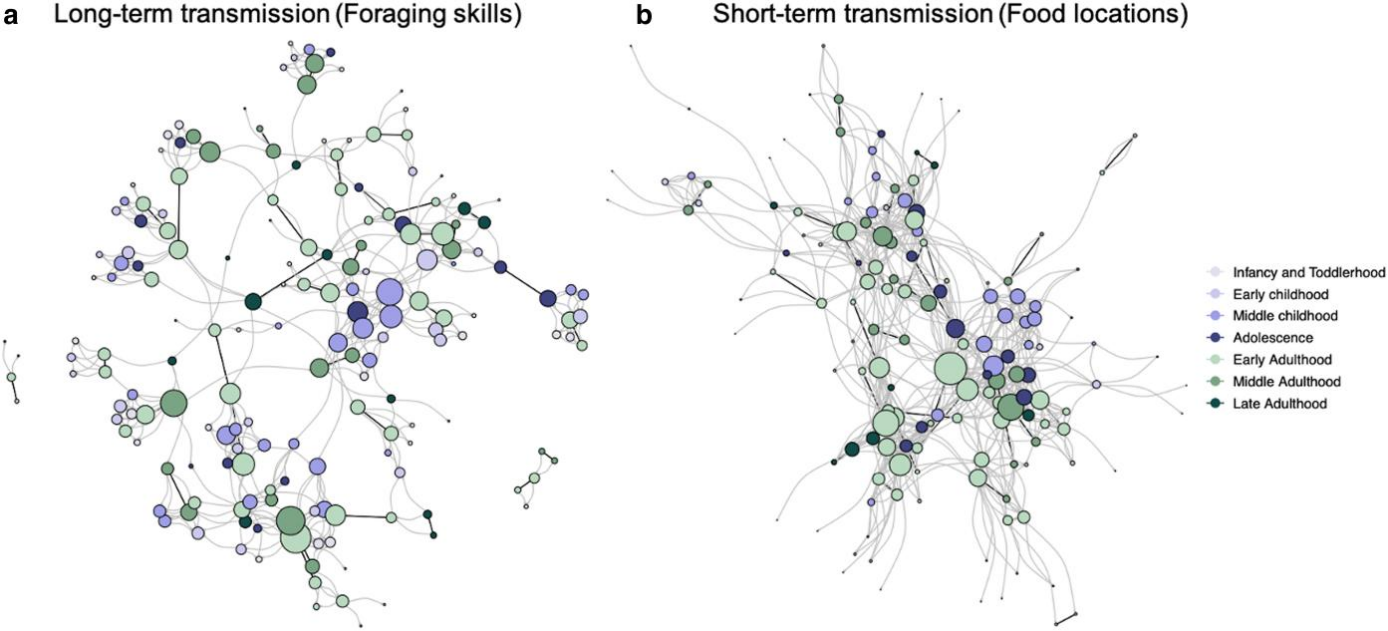
Knowledge transmission networks exhibit different structures in the contexts of long-term and short-term learning. Here, we show digraphs of a) long-term and b) short-term knowledge transmission networks. These digraphs represent our models’ predictions, with each node (i.e. circle) representing an individual and each directed tie (i.e. line) indicating predicted knowledge transmission between individuals. Nodes are sized by the number of outgoing ties and are distinguished by age class (younger to older children/adolescents; younger to older adults; see legend). Ties between spouses (bold lines) are distinguished from those between nonspouses (thin lines). a) Long-term knowledge transmission tends to cluster into smaller mixed-age units within the community, whereas b) short-term knowledge transmission is less fragmented but appears to cluster by age, with connections present between children of different ages and between adults of different ages. a) Long-term knowledge transmission (Foraging skills), and b) short-term knowledge transmission (Food locations).

Throughout the study, individuals made 1,633 nominations in our double-sampled social network questions regarding the transmission of foraging skills and food location information. We applied latent network models to analyze these data, as they allow us to estimate the individual-level (e.g. sex, age) and dyadic-level (e.g. kinship, spouseship) factors that influence the long-term and short-term learning networks, while also estimating and adjusting for individual-level tendencies to over- or under-report ties (see Ref. (66 ), for a detailed outline and validation of the models). We then extracted predicted networks from the models for long-term transmission (foraging skills; Fig. 1a) and short-term transmission (food locations; Fig. 1b). The inferred networks exhibit different structures, with the long-term transmission network having fewer nominations and being less dense ($N_{\mathrm{ties}}=408,\mathrm{density}=0.013$) than the short-term network ($N_{\mathrm{ties}}=689,density=0.021$). Alongside this, the long-term transmission network of foraging skills was characterized by very low levels of reciprocity, with only 8% of ties being reciprocated, while 45% of ties were reciprocated in the short-term transmission network of food locations (see Table S1). As shown in Fig. 1, long-term knowledge transmission tends to cluster into smaller mixed-age units within the community—which we expect to be family units—whereas short-term knowledge transmission is less fragmented but appears to cluster by age, with connections present between children of different ages (i.e. the purple nodes in the figures) and between adults of different ages (i.e. the green nodes). We now turn to our analytical results to understand why these networks are structured in this way. Unless otherwise stated, we present our results as the posterior median (*θ*) and 90% credible intervals (CI).

### Short-term learning is reciprocal, but long-term learning is asymmetric

**Fig. 2.**
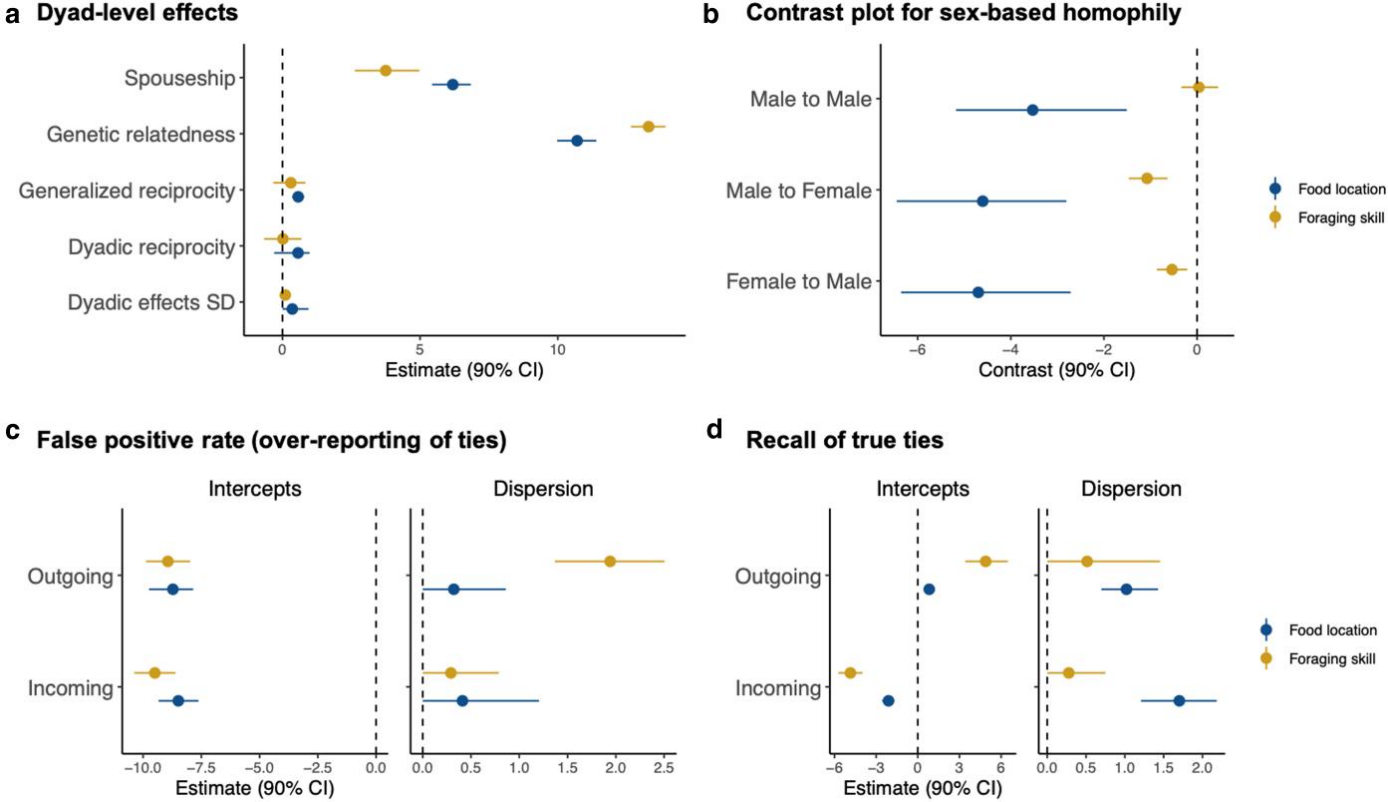
Both long-term and short-term learning are shaped by kinship, marital partnerships, and sex-based homophily, and individuals were more likely to forget from whom they acquire knowledge. We present the following: a) dyadic parameter estimates, b) a contrast plot for sex-based homophily, c) the false positive rate, and d) recall bias of true ties, in both long-term learning of foraging skills and short-term learning of food locations. a) The model baselines are based on unrelated, nonspouse dyads. b) In the contrast plot, the reference is the probability of transmitting knowledge between female-to-female dyads. The plot displays the differences between this reference (female-to-female dyads) and male-to-male, male-to-female, and female-to-male dyads. A negative coefficient, with a CI that does not overlap 0, indicates a significantly lower likelihood of knowledge transmission in male-to-male, male-to-female, and female-to-male dyads compared to female-to-female dyads. c) Individuals rarely falsely reported either incoming or outgoing ties that did not exist, across both long-term and short-term transmission networks. d) Individuals were more likely to forget incoming ties, while outgoing ties were reported more accurately. This pattern was more prominent in the transmission of foraging skills. a) Dyad-level effects, b) contrast plot for sex-based homophily, c) false positive rate (over-reporting of ties), and d) recall of true ties.

Reflecting the descriptive features of our knowledge transmission networks (see Fig. 1), our results show that long-term transmission of foraging skills does not exhibit a clear pattern of dyadic reciprocity (θ=0.018,CI=[−0.667,0.689]; see Fig. 2a). Additionally, individuals who transmitted foraging skills to many others did not reliably receive skills in return (i.e. the correlation between sending and receiving ties, often referred to as *generalized reciprocity* in the literature: θ=0.306,CI=[−0.33,0.835]). These findings suggest that individuals who share foraging skills do not necessarily receive them from the same individuals, and those who share foraging skills widely within the community are not more likely to receive them in return from various individuals. In contrast, we found mixed evidence that short-term transmission of food location information is patterned by dyadic reciprocity. After conditioning on other covariates (e.g. genetic relatedness, which might explain some reciprocity), our estimate of residual dyadic reciprocity was positive but encompassed 0 (θ=0.565,CI=[−0.3,0.986]). Individuals who shared food location information *with* many others in the community were more likely to receive food location information *from* many others (θ=0.572,CI=[0.354,0.783]), indicating a pattern of generalized reciprocity.

### The age-structure of knowledge transmission explains why long-term learning is asymmetric

**Fig. 3.**
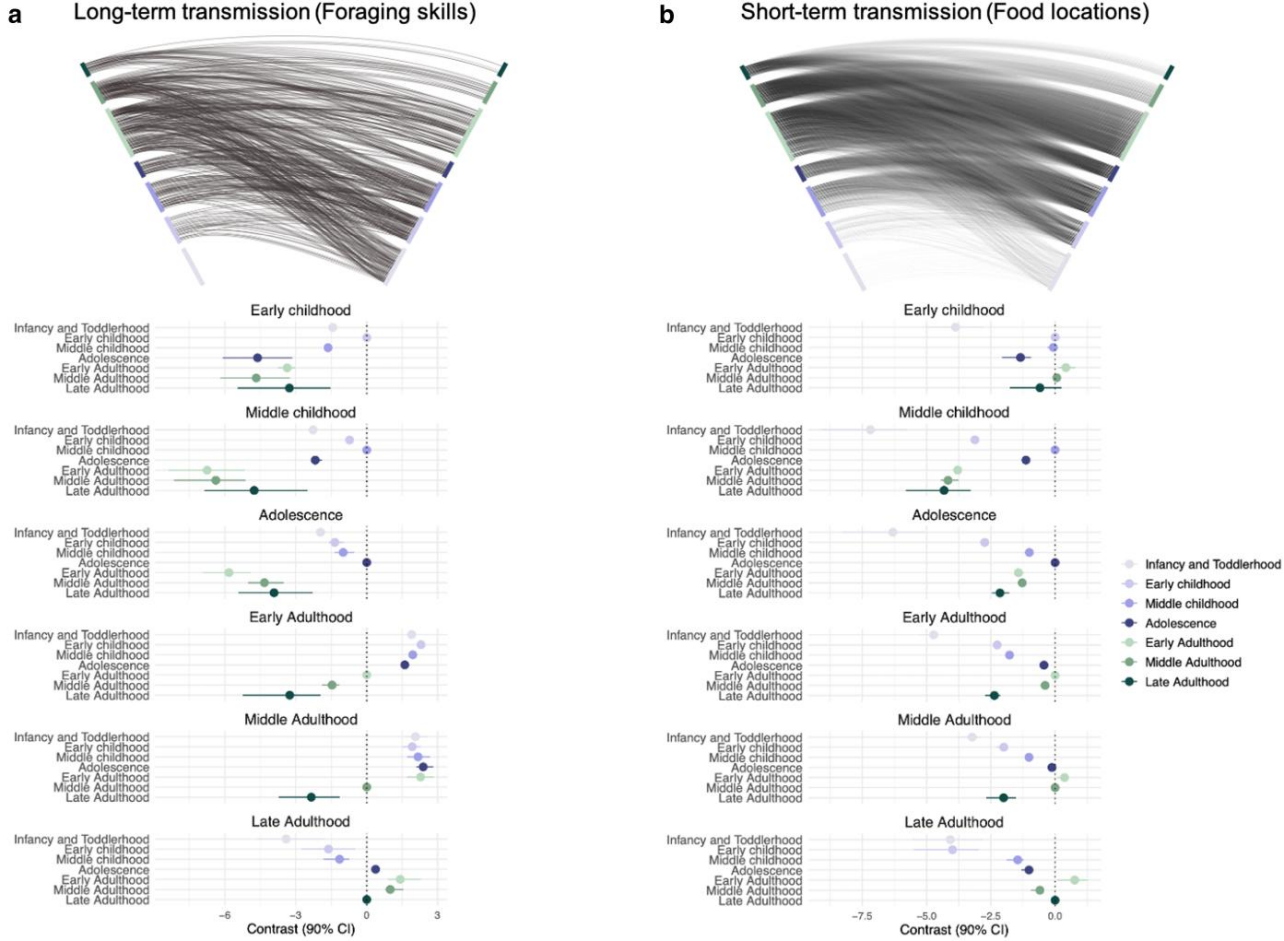
The age-structure of knowledge transmission in-part explains why long-term learning is asymmetric. Here, we show hive plots (*upper row*) and contrast plots (*lower row*) of a) long-term and b) short-term knowledge transmission, as predicted by our latent network models. In the hive plots (*upper row*), squares on the left axis represent individuals *sending knowledge*, while squares on the right represent individuals *receiving knowledge*. These individuals are colored according to their age class, with the oldest age class at the top of the figure and the youngest at the bottom. The lines connecting individuals represent reported events of knowledge transmission ties. In the contrast plots (*lower row*), the reference is the probability of transmitting knowledge between peers (within-class ties) in each age-class category. The plots display the differences between the reference (within-class ties) and between-class ties (combinations of focal age-class and other age-classes). A positive coefficient, with a CI not overlapping 0, represents a robust higher likelihood of ties between age-classes than between peers, while a negative coefficient would suggest the opposite. a) Long-term knowledge transmission (Foraging skills), and b) short-term knowledge transmission (Food locations).

Figure 3 displays the results related to age class. While our analyses provide rich insights into how knowledge transmission is structured by age, we will focus on the most important findings here. Our results suggest that individuals, in general, tend to share foraging skills with people who are the same age or younger (see Fig. 3a upper row). A similar pattern is observed with food location ties, which are denser in terms of the number of connections (see Fig. 3b upper row).

The age structure of long-term foraging skill transmission (shown in the contrast plot in Fig. 3a lower row) mirrors the pattern observed in the hive plot (Fig. 3a upper row): individuals generally share foraging skills with those who are the same age or younger. Specifically, children in early childhood are most likely to transmit foraging skills to their peers in early childhood. Similarly, those in middle childhood and adolescence are more likely to share foraging skills with their peers and younger children. Individuals in early adulthood tend to transmit foraging skills to children across all age classes, providing evidence of both vertical and oblique transmission of foraging skills. A similar pattern is observed for individuals in middle adulthood, who are more likely to share foraging skills with younger age classes and less likely to share with those in late adulthood, compared to sharing with their peers in middle adulthood. Interestingly, individuals in late adulthood are more likely to share foraging skills with younger *adults* but less likely to share with younger children. This suggests that learning foraging skills continues throughout adulthood, with older individuals still passing on their knowledge to younger adults.

However, we observed different age structuring in the short-term transmission of food location information (Fig. 3b lower row). Children in early childhood share food location information with individuals from different age groups, including those in early adulthood and middle adulthood—likely their parents—as well as peers in the same age class and older children in middle childhood. In contrast, as observed in the foraging skill transmission networks, children in middle childhood and adolescence tend to share food location information primarily with peers in their own age class, likely because they often forage in peer groups. Adults in early and middle adulthood are more likely to share food location information with peers in their own age class, as well as with adolescents and other adults in similar age classes. Individuals in late adulthood are most likely to share food location information with individuals in early adulthood, who are likely their offspring.

In sum, the age structure of long-term transmission networks suggests that foraging skills are more commonly shared from older to younger individuals, which explains the asymmetry observed in long-term learning (Fig. 2a). In contrast, short-term transmission networks are less patterned by hierarchical age structures and are more shaped by reciprocal interactions among peers or individuals in similar age groups, which accounts for the generalized reciprocity observed in food location information sharing (Fig. 2a).

### Both short-term and long-term learning occur between spouses and kin

As shown in Fig. 2a, both long-term transmission (θ=13.299,CI=[12.655,13.912]) and short-term transmission (θ=10.703,CI=[9.976,11.396]) are much more likely to occur between kin. Spouses exchange information about food locations (θ=6.187,CI=[5.434,6.841]). Although less frequent than the sharing of food location information, spouses also share foraging skills with one another (θ=3.751,CI=[2.631,4.976]).

### Knowledge transmission is structured by sex-based homophily

Here, we present contrast coefficients (*Δ*) and 90% CI. The contrast coefficients represent the difference in the likelihood of observing ties between dyadic sex combinations, using ties between women (i.e. “female to female”) as a reference. As shown in Fig. 2b, we observe a negative *Δ*, with a CI that does not overlap 0, for dyads of different sexes (i.e. “male-to-female,” “female-to-male”) in both the food location information (*navy circles*) and foraging skill (*orange circles*) transmission networks. Dyads between men (i.e. “male to male”) in food location information transmission networks (*navy circles*) have a negative *Δ* (Δ=−3.529,CI=[−5.175,−1.509]). In contrast, the *Δ* for sharing foraging skills between males centers around zero (Δ=0.038,CI=[−0.336,0.450]), indicating that males share foraging skills with other males just as frequently as females share them with other females. Taken together, these results suggest a lower likelihood of both long-term foraging skill and short-term food location information transmission occurring between different sexes, and that females are most likely to share food location information with other females.

### Individuals forget from whom they receive knowledge

Our latent network models incorporate a measurement model that explicitly estimates and adjusts for measurement biases typically found in self-reported social network data. For a detailed outline of the model, see Ref. (66). This estimation of individuals’ biases in reporting who they give and/or receive long-term and short-term knowledge transmission is made possible as we have collected double-sampled network data—where individuals report both sides of the directed knowledge transmission ties. This allows us to obtain information about each tie from both the sender’s and receiver’s perspectives. Given this approach, we assessed whether individuals tend to provide discordant reports about the ties they are involved in, compared to the reports of others involved in the same ties. We further estimated the average tendencies across our sample, such as the likelihood of reporting ties that do not exist or forgetting to report ties that do exist. As shown in Fig. 2c, our results indicate that, on average, individuals rarely falsely reported either incoming or outgoing ties that did not exist, across both long-term and short-term transmission networks. There was also little variation across individuals in terms of false reporting. Importantly, we found that individuals, on average, were *more likely to forget who shared foraging skills with them* (i.e. incoming ties), while they *reported more accurately with whom they shared foraging skills* (i.e. outgoing ties) (Fig. 2d). We observed similar patterns of bias in the transmission of food location information, although the bias was smaller and more variable across individuals.

## Discussion

The present study provides important insights into the structure of cultural transmission networks within a BaYaka forager community, highlighting key distinctions between long-term, skill-intensive learning and short-term, information-based learning processes. Our findings reveal that both types of learning are structured by kinship, sex-based homophily, and marital partnerships, but differ in network density, age patterning, and reciprocity. Specifically, foraging skills are transmitted asymmetrically—from experienced, older individuals to younger ones—along with horizontal transmission occurring among peers during childhood (Fig. 3a). These results align with theories of cultural evolution that emphasize the value of skill accumulation and inter-generational knowledge transfer (12). In contrast, the transmission of food location information is broader and more reciprocal, especially among peers, and this horizontal transmission extends from childhood through to adulthood (Fig. 3b). In particular, we found that young children share food location information across all age groups, interacting with both peers and older individuals (Fig. 3b), suggesting that immediate, adaptive information is shared more fluidly across ages. These findings support the theory that, in rapidly changing environments, younger individuals serve as crucial sources of up-to-date knowledge and information (10), highlighting the role of children as key agents of cultural adaptation (67). Below, we interpret these age-based differences in knowledge-sharing networks through the lens of cultural evolution theory, emphasizing how these patterns reflect demographic factors that shape diverse learning processes within a population.

Our findings show that long-term and short-term learning networks exhibit distinct structures, reflecting the differing opportunity costs associated with each type of learning. For example, teaching and learning specific skills needed to efficiently extract wild yams or collect honey (i.e. long-term learning) require considerably more time and energy than sharing information about the location of a tree with ripe fruit that someone has spotted (i.e. short-term learning). Therefore, foraging skills—which are costly and time-intensive to teach and learn—are typically shared within smaller, close-knit networks with lower reciprocity, often among close kin and peer groups. While the transmission of foraging skills often follows age-structured pathways from experienced elders to younger individuals, our results highlight the critical role of peer learning from early childhood to adolescence. This pattern aligns with theories in cultural evolution that predict children acquire skills from elders through vertical (parent-to-child) and oblique (from older nonparent adults) transmission, as well as from peers through horizontal (peer-to-peer) transmission during play and practice (12, 15). Thus, our results reinforce the idea that while learning from older individuals is crucial for acquiring complex foraging skills, horizontal transmission among peers is also an essential mechanism of skill development in childhood. This supports theoretical models and empirical studies on optimal learning processes and peer-based skill exchange (see Refs. (67–69)).

Crucially, our findings extend the multistage learning model (15) beyond childhood, demonstrating the continued relevance of the theory in understanding learning throughout adulthood. Previous studies with Tsimane horticulturalists in Bolivia have shown that reproductive-aged adults continue to learn about food resources—primarily to meet the demands of their offspring—and that postreproductive adults possess the most extensive knowledge (22, 34, 70). Our results support this finding and provide further empirical evidence that older, postreproductive individuals in late adulthood pass down their expertise to younger adults in early and middle adulthood, who then transmit these foraging skills to younger generations. This highlights the role of postreproductive individuals in cultural transmission, which has not received much attention (see Ref. (13)). This inter-generational transmission also suggests that adults not only continue to learn from experienced elders but also play a crucial bridging role in facilitating the flow of knowledge between children and the older generation. This bridging role mirrors patterns observed in middle childhood, where individuals in middle childhood seem to facilitate knowledge transfer between younger children and adolescents (see Ref. (55)). Such patterns emphasize the applicability of the multistage learning model across the lifespan, supporting the idea that skill acquisition in foraging societies is a lifelong, cumulative process shaped by inter-generational learning. Therefore, these findings shed light on how the need for and development of embodied capital (i.e. an individual’s physical and cognitive abilities) persist into adulthood. This expands the scope of embodied capital theory—which primarily focused on explaining the extended childhood period (19)—and raises new questions about how this continuous learning throughout adulthood may shape human life history more broadly.

In contrast to age-structured transmission of foraging skills, food location information is shared horizontally among peers, both in childhood and adulthood (Fig. 3b). This highlights the important role of peer interactions, not only in practicing and learning foraging skills during childhood but also in the continuous exchange of information throughout adulthood. Such exchanges are likely driven by reciprocity, reflecting the collaborative nature of subsistence activities in foraging societies like the BaYaka. In societies where foraging returns vary from day to day, individuals share resources (e.g. food or information) with community members based on reciprocity to buffer the risks of food scarcity (71, 72). Our findings—that individuals who share information with many others also receive food location information in return from many others—suggest that these reciprocal exchanges foster trust and cooperation within the community. Notably, we found differences in partner preferences for sharing food location information between early childhood and other age groups. Compared to other age groups—such as those from middle childhood to adulthood who exhibit a strong preference for horizontal transmission among peers—young children exchange food location information more broadly, interacting with both peers and older individuals, including parents and grandparents. This reflects that in early childhood, children interact with a larger number of community members compared to middle childhood and adolescence, likely due to the cooperative childcare they receive (73). Such broader interactions in early childhood with a wide range of individuals further support the idea that the intimate living conditions of hunter–gatherer societies facilitate cultural knowledge to be embodied in children, creating opportunities for cumulative transmission, as recently proposed by Hewlett et al. (13). As children grow older, they tend to narrow their choice of partners for skill and information sharing, which likely strengthens reciprocal bonds and mutual trust within peer groups, thereby fostering cooperative relationships that can persist into adulthood.

Our ethnographic observations provide further insight into this pattern. Young BaYaka children often join their mothers in mixed-age foraging groups, compared to children in middle childhood and adolescence who go foraging mostly in peer groups. With their small body size and fewer responsibilities for food collection, young children have the freedom to explore forest areas and often venture into spots that are less accessible to adults, while adults are more focused on gathering food and tend to stay on established walking trails. Upon spotting food sources, however, young children typically share this information with others—including older children and adults—rather than attempting to collect the food themselves, due to their limited access to tools and insufficient physical strength or foraging skills. Previous research has acknowledged that, even when young children lack the skills for full participation, they support adult foraging efforts—such as by providing childcare to infants while mothers are foraging (74, 75). Our findings reveal another way children contribute to subsistence activities: by sharing food location information that they discover. This aligns with the pooled energy budget model, which emphasizes that all group members contribute resources and energy to a shared pool based on their age and abilities (76). Our findings provide empirical evidence of how young children contribute to the community’s pooled energy budget and highlight the conditions that facilitate knowledge transmission by younger individuals.

Our results further emphasize the role of kinship, sex, and marital partnerships in shaping knowledge-sharing networks (Fig. 2a). Kin-based transmission aligns with theories of parental teaching and cooperation among relatives due to shared genetic interests (47, 77). Sex-based homophily also shapes knowledge exchange, with foraging skills typically shared within the same sex due to the sexual division of labor (61, 64). Notably, sex-segregated patterns shift when it comes to sharing food location information: women frequently share such information with each other, while men are less likely to share among themselves, reflecting BaYaka women’s collaborative group foraging (55) compared to men’s often solitary hunting (78, 79). Marital partnerships also play a crucial role in knowledge transmission. Spouses share food location information with each other that is critical for family provisioning. For example, BaYaka women share honey-tree locations with their husbands after spotting them during group foraging with other women, and couples subsequently collect honey together. Similarly, BaYaka men inform their wives about wild yam patches that they encountered while hunting. Beyond sharing such information, spouses also exchange foraging skills, which can be particularly important when one partner relocates to the other’s natal village and must adapt to local foraging techniques (e.g. pond fishing techniques (80 )). This dynamic not only supports a key assumption of cumulative cultural evolution by facilitating the acquisition of locally relevant skills (2, 3), but it also aligns with a recent theoretical model highlighting the importance of postmarital transmission (81). Together, these demographic factors, in addition to the age structuring of knowledge-sharing networks, serve an adaptive function by optimizing learning efficiency and guiding individuals to appropriate knowledge sources based on their life stage and learning needs.

Lastly, our findings suggest a cognitive bias in knowledge transmission: individuals tend to forget some of the sources from which they acquire knowledge but more accurately recall the recipients of the knowledge they share (Fig. 2d). This pattern suggests a stronger awareness of one’s role in actively sharing knowledge with others, rather than receiving it, which may shape network dynamics in both long- and short-term contexts. These findings highlight the importance of understanding individual perceptions of learning and underscore the need for caution when measuring and modeling social learning in real-world settings. Mixed-methods approaches—combining observational data, double-sampled interviews that assess ties from both the sender’s and receiver’s perspectives, and carefully constructed measurement models that account for relevant biases—can enhance inferences about transmission dynamics. By addressing these biases, our study was able to capture directionality-based perception errors, supporting recent cultural evolution theory that suggests fallible memory can influence learning strategies (11). Our findings open fruitful avenues for future theoretical and empirical research to further explore how perception may bias learning and how these biases may impact cultural evolutionary dynamics.

In conclusion, our study provides empirical insights into cultural transmission within a foraging society, emphasizing how knowledge is shared through age-specific pathways that differentiate long-term and short-term learning. We also highlight the role of demographic factors such as sex, kinship, and marital ties in shaping these knowledge-sharing dynamics. Our findings demonstrate how the costs and time required to acquire knowledge influence the structure of these networks, supporting theoretical models of cultural evolution that link learning strategies to the type of knowledge being shared (9 , 10). This dynamic optimizes learning efficiency by directing knowledge through the most relevant social connections. Furthermore, we demonstrate that cultural learning extends into adulthood, emphasizing the importance of lifelong learning in adapting to changing environments. In sum, our findings shed light on how a society’s foraging culture is reflected in its knowledge transmission networks, which play a key role in facilitating human adaptation to diverse environments through both long-term, stable learning and short-term, flexible learning processes. In doing so, this study offers valuable evidence of the mechanisms that support human resilience and cumulative cultural evolution in real-world contexts.

## Methods

### Ethics and inclusion statement

Permissions to conduct research in the Republic of the Congo were obtained from the Institut de Recherche en Sciences Exactes et Naturelles (IRSEN) and the Institut National de Recherche en Sciences Sociales et Humaines (INRSSH) in Brazzaville. Our study procedures and methods were carried out in accordance with the national laws, as well as the relevant guidelines and regulations of the Republic of the Congo. The study protocol was approved by the Institut National de Recherche en Sciences Sociales et Humaines (approval no: 007/MESRSIT/INRSSH-DG) and by the Max Planck Society’s Ethics Council in Germany (application no: 2022_7). H.J. presented an overview of the project, including the research aim and methods, at a public meeting with all the inhabitants of the study village and obtained informed consent from the community. Consent from each inhabitant was obtained after the meeting, while collecting basic demographic and household-level data. All interview data and individual information were anonymized.

### Data collection

The data presented in this manuscript were collected between August and October in 2022, in collaboration with a Bayaka community in a village along the Motaba River in the Likouala Department, the Republic of the Congo. H.J. collected data in a semi-sedentary village with seasonal mobility throughout the year due to trade, marriage, and subsistence activities (e.g. fishing seasons). Residents maintain their homes in the village during visits to other villages or forest camps, but return to the village, where they also have small crop fields. At the beginning of the study period, we conducted a household survey for all households in the village (N=35) and collected demographic data for all residents, including their sex and age class. The village census counted 181 residents, a mix of kin and nonkin family members. Age classes were defined as follows: infancy and toddlerhood (from 0 to 3 years; N=36), early childhood (from 4 to 6 years; N=25), middle childhood (from 7 to 13 years; N=28), adolescence (from 14 to 19 years; N=13), early adulthood (from 20 to 39 years; N=48), middle adulthood (from 40 to 59 years; N=20), and late adulthood (≥60 years; N=11). We also collected marital data and observed 32 marriages within the community (referred to as spouses throughout the manuscript). Kinship relationships were determined through genealogical interviews, and genetic relatedness (i.e. Wright’s coefficient of relatedness: (82)) was calculated among all residents using the kinship2 package (v.1.8.5; (83)).

### Long-term and short-term knowledge transmission network interviews

To collect data on the full knowledge transmission networks, we conducted self-reported “name generator” interviews with all 132 inhabitants in the study village (66 females, 66 males) who were older than 4 years (aged from 4 to 75). Our network data were “double sampled”, meaning that we asked each individual about both directions of their knowledge transmission ties. This approach allows us to gather perspectives on the same directed ties from both individuals involved (84 ). For long-term knowledge transmission, we asked individuals, “*Who did you share foraging skills with?*” and then “*Who shared foraging skills with you?*”. For short-term knowledge transmission, we asked, “*Who did you share food location information with?*” and then “*Who shared food location information with you?*”. Interviewees freely listed individuals, not limited to village residents, until they indicated that there were no more to name (i.e. there was no upper limit to the number of individuals that could be nominated). However, for the analyses, nonresidents (N=21) and deceased individuals (N=26) were excluded to create complete community-wide networks.

### Latent network models

To analyze the structure of our knowledge transmission networks, we applied a bespoke latent network model that combines a sub-model for estimating the social network structure—integrating the social relations model (85) and the stochastic blockmodel (86)—with a measurement model that estimates and adjusts for the measurement biases typically present in “name generator” data (see Ref. (87, 88), for recent discussions of measurement biases). For a full outline of the model structure, prior specifications, and exhaustive model validation, see Ref. (66). Within the latent network model, we specified parameters that capture variation at the *individual level*, including parameters for the variation in the number of ties individuals send and receive, as well as the correlation between sending and receiving ties (referred to here as generalized reciprocity). To examine how knowledge transmission networks were structured at the *dyadic level*, we included parameters for dyadic reciprocity, as well as the dyad-level effects that capture whether individuals were more likely to share knowledge with those (i) who were genetically related to themselves and (ii) who were their marital partners. We further included age class and sex as known grouping variables in our stochastic block submodels to directly test our predictions regarding the age- and sex-based structure of knowledge transmission. This allowed us to estimate the probability of knowledge transmission both within and between each age class and sex. To estimate and adjust for the measurement biases associated with self-reported network data, we included parameters that capture individual tendencies to erroneously report ties that do not exist, to forget ties that do exist, and to duplicate reports across the double sampled questions (which were always asked in the same order). All analyses were conducted in R (89). The latent network models were coded in “cmdstanr” (90, 91) and fitted using the “STRAND” package (66, 92), with two chains, 2,000 warm-up iterations, and 4,000 sampling iterations. We diagnosed model fit and Markov Chain Monte Carlo performance using trace plots, $\hat{R}$, and reported effective sample sizes. All diagnostics indicated good model fit.

## Supplementary Material

pgaf258_Supplementary_Data

## Data Availability

All relevant data and code for reproducing the analyses and figures are available at the following GitHub repository: https://github.com/haneuljangkr/bayaka-knowledge-transmission.

## Preprints

A preprint of this article (93) was posted on the Open Science Framework preprint server (OSF) and can be found at: https://osf.io/preprints/osf/25c3x.
