# Supplementary material for: Transmission networks of long-term and short-term knowledge in a foraging society
Source: PNAS Nexus. 2025 Sep 8;4(9):pgaf258. doi: 10.1093/pnasnexus/pgaf258 (PMC12415857; doi:10.1093/pnasnexus/pgaf258)
Supplement: pgaf258_Supplementary_Data [file pgaf258_supplementary_data.pdf]

Supplementary information for "Transmission Networks of Long-term and Short-term Knowledge in a Foraging Society"

Haneul Jang[a,b,1] and Daniel Redhead[b,c,d]

[a] *Institute for Advanced Study in Toulouse, Toulouse School of Economics, 1 Esplanade de l'Université, 31080 Toulouse cedex 06, France.*
[b] *Department of Human Behavior, Ecology and Culture, Max Planck Institute for Evolutionary Anthropology, Deutscher Platz 6, 04103 Leipzig, Germany.*
[c] *Department of Sociology, University of Groningen, Grote Rozenstraat 31, 9712 TG Groningen, The Netherlands.*
[d] *Inter-University Center for Social Science Theory and Methodology, University of Groningen, Groningen, The Netherlands.*

Table S1. Network descriptives of long-term transmission of foraging skills and short-term transmission of food locations. The inferred networks represent the extracted predicted networks from the latent network models.

Type	N_ties	Density	Reciprocity	Transitivity	Centralization	Mean degree	In-degree	Out-degree
**Foraging skills**								
**inferred**	408	0.013	0.083	0.431	0.024	2.254	0 - 10	0 - 8
**give**	430	0.013	0.079	0.379	0.037	2.376	0 - 18	0 - 8
**receive**	230	0.007	0.078	0.325	0.049	1.271	0 - 15	0 - 8
**Food locations**								
**inferred**	686	0.021	0.449	0.365	0.077	3.79	0 - 17	0 - 18
**give**	556	0.017	0.417	0.349	0.058	3.072	0 - 16	0 - 14
**receive**	417	0.013	0.321	0.334	0.057	2.304	0 - 18	0 - 10

Table S2. Results of dyadic effects and measurement of false positive rate and recall biases of true ties in long-term transmission of foraging skills and short-term transmission of food locations

Network	Type	Variable	Median	LowerCI	UpperCI
**Foraging skills**	Dyadic Effects	Genetic relatedness	13.299	12.655	13.912
		Spouseship	3.751	2.631	4.976
		Generalized reciprocity	0.306	-0.33	0.835
		Dyadic reciprocity	0.018	-0.667	0.689
		Dyadic effects SD	0.103	0	0.249
	False Positive Rate	intercept (outgoing)	-8.933	-9.876	-7.98
		intercept (incoming)	-9.492	-10.376	-8.61
		SD (outgoing)	1.941	1.369	2.504
		SD (incoming)	0.292	0.001	0.788
	Recall of True Ties	intercept (outgoing)	4.888	3.428	6.482
		intercept (incoming)	-4.851	-5.721	-3.978
		SD (outgoing)	0.514	0	1.454
		SD (incoming)	0.278	0.002	0.749
	Sender Effects	Sender effects SD	0.698	0.493	0.928
	Receiver Effects	Receiver effects SD	0.147	0.001	0.313
**Food locations**	Dyadic Effects	Genetic relatedness	10.703	9.976	11.396
		Spouseship	6.187	5.434	6.841
		Generalized reciprocity	0.572	0.354	0.783
		Dyadic reciprocity	0.565	-0.3	0.986
		Dyadic effects SD	0.357	0.002	0.947
	False Positive Rate	intercept (outgoing)	-8.719	-9.743	-7.846
		intercept (incoming)	-8.485	-9.335	-7.622
		SD (outgoing)	0.322	0	0.861
		SD (incoming)	0.412	0.001	1.204
	Recall of True Ties	intercept (outgoing)	0.824	0.538	1.133
		intercept (incoming)	-2.107	-2.57	-1.697
		SD (outgoing)	1.021	0.697	1.428
		SD (incoming)	1.702	1.208	2.183
	Sender Effects	Sender effects SD	0.844	0.658	1.004
	Receiver Effects	Receiver effects SD	0.721	0.56	0.864

Table S3. Results of gender block effects in long-term transmission of foraging skills and short-term transmission of food locations

Network	Variable	Median	LowerCI	UpperCI
**Foraging skills**	Female to Female	-3.715	-5.875	-1.948
	Female to Male	-4.27	-6.079	-2.094
	Male to Female	-4.798	-6.63	-2.649
	Male to Male	-3.676	-5.611	-1.662
**Food locations**	Female to Female	-2.509	-4.437	-0.677
	Female to Male	-4.698	-6.38	-2.635
	Male to Female	-4.603	-6.491	-2.739
	Male to Male	-3.529	-5.259	-1.474

Table S4. Results of age block effects in long-term transmission of foraging skills

Network	Sender Type	Receiver Type	Median	LowerCI	UpperCI
**Foraging skills**	Infancy and Toddlerhood	Infancy and Toddlerhood	-8.253	-10.437	-6.276
		Early childhood	-9.001	-11.712	-6.82
		Middle childhood	-8.679	-11.265	-5.996
		Adolescence	-8.127	-10.838	-5.465
		Early Adulthood	-9.521	-12.027	-7.411
		Middle Adulthood	-8.625	-11.498	-6.326
		Late Adulthood	-7.356	-10.268	-4.331
	Early childhood	Infancy and Toddlerhood	-5.318	-6.403	-4.397
		Early childhood	-3.879	-4.901	-2.838
		Middle childhood	-5.513	-6.561	-4.384
		Adolescence	-8.496	-10.986	-5.987
		Early Adulthood	-7.24	-8.672	-5.864
		Middle Adulthood	-8.55	-11.087	-6.089
		Late Adulthood	-7.149	-10.353	-4.367
	Middle childhood	Infancy and Toddlerhood	-4.687	-5.733	-3.627
		Early childhood	-3.137	-3.986	-2.206
		Middle childhood	-2.416	-3.289	-1.555
		Adolescence	-4.592	-5.611	-3.449
		Early Adulthood	-9.163	-11.67	-6.732
		Middle Adulthood	-8.797	-11.438	-6.683
		Late Adulthood	-7.175	-10.149	-4.05
	Adolescence	Infancy and Toddlerhood	-4.71	-5.98	-3.426
		Early childhood	-4.101	-5.218	-2.934
		Middle childhood	-3.749	-4.773	-2.692
		Adolescence	-2.751	-4.246	-1.333
		Early Adulthood	-8.576	-11.18	-6.225
		Middle Adulthood	-7.075	-9.268	-4.849
		Late Adulthood	-6.671	-9.672	-3.626
	Early Adulthood	Infancy and Toddlerhood	-3.211	-3.993	-2.426
		Early childhood	-2.819	-3.599	-1.971
		Middle childhood	-3.167	-3.984	-2.363
		Adolescence	-3.501	-4.398	-2.454
		Early Adulthood	-5.113	-5.896	-4.2
		Middle Adulthood	-6.578	-7.789	-5.36
		Late Adulthood	-8.368	-11.145	-6.143
	Middle Adulthood	Infancy and Toddlerhood	-3.423	-4.34	-2.478
		Early childhood	-3.557	-4.599	-2.579
		Middle childhood	-3.311	-4.2	-2.373
		Adolescence	-3.093	-4.064	-1.996
		Early Adulthood	-3.204	-4.038	-2.385
		Middle Adulthood	-5.482	-6.879	-4.09
		Late Adulthood	-7.826	-10.597	-5.228
	Late Adulthood	Infancy and Toddlerhood	-7.269	-10.248	-4.175
		Early childhood	-5.475	-8.379	-2.876
		Middle childhood	-5.009	-7.429	-3.147
		Adolescence	-3.485	-5.176	-1.942
		Early Adulthood	-2.443	-3.323	-1.505
		Middle Adulthood	-2.866	-4.048	-1.632
		Late Adulthood	-3.86	-5.604	-2.406

Table S5. Results of age block effects in short-term transmission of food locations

Network	Sender Type	Receiver Type	Median	LowerCI	UpperCI
**Food locations**	Infancy and Toddlerhood	Infancy and Toddlerhood	-8.705	-10.831	-6.468
		Early childhood	-8.401	-10.192	-6.746
		Middle childhood	-9.068	-11.504	-6.698
		Adolescence	-8.51	-11.177	-6.131
		Early Adulthood	-7.513	-8.706	-6.393
		Middle Adulthood	-8.991	-11.496	-6.714
		Late Adulthood	-8.104	-10.871	-5.601
	Early childhood	Infancy and Toddlerhood	-9.668	-12.023	-7.344
		Early childhood	-5.799	-7.002	-4.535
		Middle childhood	-5.864	-7.017	-4.831
		Adolescence	-7.143	-9.063	-5.464
		Early Adulthood	-5.378	-6.206	-4.412
		Middle Adulthood	-5.733	-6.964	-4.61
		Late Adulthood	-6.387	-8.765	-4.279
	Middle childhood	Infancy and Toddlerhood	-9.169	-11.876	-6.924
		Early childhood	-5.104	-6.063	-4.182
		Middle childhood	-1.988	-2.793	-1.151
		Adolescence	-3.12	-3.993	-2.195
		Early Adulthood	-5.775	-6.75	-4.811
		Middle Adulthood	-6.15	-7.236	-4.904
		Late Adulthood	-6.305	-8.593	-4.429
	Adolescence	Infancy and Toddlerhood	-8.512	-11.539	-6.286
		Early childhood	-4.938	-6.141	-3.81
		Middle childhood	-3.202	-4.202	-2.341
		Adolescence	-2.203	-3.245	-1.225
		Early Adulthood	-3.629	-4.436	-2.706
		Middle Adulthood	-3.481	-4.429	-2.381
		Late Adulthood	-4.347	-5.703	-3.003
	Early Adulthood	Infancy and Toddlerhood	-7.552	-8.826	-6.564
		Early childhood	-5.079	-5.891	-4.213
		Middle childhood	-4.599	-5.495	-3.816
		Adolescence	-3.26	-4.077	-2.394
		Early Adulthood	-2.828	-3.552	-2.03
		Middle Adulthood	-3.215	-4.03	-2.459
		Late Adulthood	-5.19	-6.277	-4.153
	Middle Adulthood	Infancy and Toddlerhood	-6.894	-8.451	-5.486
		Early childhood	-5.665	-6.896	-4.556
		Middle childhood	-4.687	-5.614	-3.68
		Adolescence	-3.795	-4.743	-2.741
		Early Adulthood	-3.305	-4.065	-2.498
		Middle Adulthood	-3.672	-4.614	-2.585
		Late Adulthood	-5.673	-7.296	-4.104
	Late Adulthood	Infancy and Toddlerhood	-7.997	-10.774	-5.185
		Early childhood	-7.911	-10.73	-5.336
		Middle childhood	-5.374	-7.116	-3.577
		Adolescence	-4.937	-6.544	-3.377
		Early Adulthood	-3.158	-3.974	-2.279
		Middle Adulthood	-4.511	-5.769	-3.311
		Late Adulthood	-3.922	-5.239	-2.371
